# Impact of medical oncologist certification on survival outcomes in metastatic colorectal cancer: evidence from the SCRUM-Japan MONSTAR-SCREEN Observational Study

**DOI:** 10.1007/s10147-026-03069-0

**Published:** 2026-06-06

**Authors:** Kyosuke Seguchi, Tadayoshi Hashimoto, Takao Fujisawa, Taro Shibuki, Chigusa Morizane, Norio Nonomura, Hiroji Iwata, Hidemichi Watari, Susumu Okano, Kenjiro Namikawa, Yoichi Naito, Hideaki Bando, Takayuki Yoshino

**Affiliations:** 1 Department of Gastroenterology and Gastrointestinal Oncology, National Cancer Center Hospital East, 6-5-1 Kashiwanoha, Kashiwa, Chiba 277-8577 Japan; 2 Translational Research Support Office, National Cancer Center Hospital East, Kashiwa, Japan; 3 Department of Head and Neck Medical Oncology, National Cancer Center Hospital East, Kashiwa, Japan; 4 Department of Hepatobiliary and Pancreatic Oncology, National Cancer Center Hospital East, Kashiwa, Japan; 5 Department of Hepatobiliary and Pancreatic Oncology, National Cancer Center Hospital, Tokyo, Japan; 6 Department of Urology, Graduate School of Medicine, Osaka University, Suita, Osaka Japan; 7 Department of Advanced Clinical Research and Development, Nagoya City University, Nagoya, Japan; 8 Department of Obstetrics and Gynecology, Hokkaido University School of Medicine, Hokkaido University, Sapporo, Japan; 9 Department of Dermatologic Oncology, National Cancer Center Hospital, Tokyo, Japan; 10 Department of Medical Oncology, National Cancer Center Hospital East, Kashiwa, Japan

**Keywords:** Medical oncologist, Board certification, Colorectal cancer, Chemotherapy, Targeted therapy, Guideline adherence

## Abstract

**Background:**

Board certification in surgical oncology improves cancer outcomes, but the impact of medical oncologist certification on systemic therapy outcomes is unknown. We investigated whether the Japanese Society of Medical Oncology (JSMO) board certification of the enrolling physician was associated with treatment quality and survival.

**Methods:**

This retrospective cohort study used data from the SCRUM-Japan MONSTAR-SCREEN observational study, a nationwide multicenter genomic screening program enrolling patients from 2019 to 2022. Standard treatment implementation rates were compared across cancer types as a secondary outcome. The primary outcome was overall survival (OS) in patients with metastatic colorectal cancer (mCRC) using Kaplan–Meier analysis and Cox proportional hazards regression with sequential adjustment for clinical and molecular covariates.

**Results:**

Among 2,184 patients, standard treatment rates were comparable between board-certified and non–board-certified groups (97.0% vs. 96.9%). In 358 patients with mCRC, enrollment by a board-certified physician was associated with longer OS (median, 36.5 vs. 30.7 months; hazard ratio (HR): 0.66, 95% CI 0.50–0.87; log-rank *p* = 0.003) even after adjustment for age, sex, tumor sidedness, region, trial participation, molecular targeted therapy, metastatic organ count (HR, 0.73; 95% CI 0.55–0.97; *p* = 0.029), and after additional adjustment for RAS and BRAF V600E status (HR, 0.64; 95% CI 0.47–0.88; *p* = 0.005). Board-certified physicians used molecular targeted therapy more frequently (89.5% vs. 80.4%; *p* = 0.036).

**Conclusions:**

JSMO board certification was independently associated with improved OS in mCRC despite comparable guideline adherence, suggesting that specialist expertise extends beyond treatment selection.

**Supplementary Information:**

The online version contains supplementary material available at 10.1007/s10147-026-03069-0.

## Introduction

The relationship between physician expertise and cancer treatment outcomes has been extensively investigated in surgical oncology. Systematic reviews have demonstrated that surgeon specialization is one of the most important determinants of superior outcomes across multiple cancer types [1–4]. In Japan, board certification systems established by surgical subspecialty societies have been associated with improved short-term outcomes after esophagectomy and higher survival rates in cervical cancer [5, 6]. These findings have provided a rationale for subspecialty certification programs as a mechanism for quality assurance in cancer care.

However, the impact of board certification in medical oncology on treatment outcomes remains largely unexamined. Unlike surgical procedures, where technical proficiency is directly linked to operative outcomes, the effect of medical oncologist certification on systemic therapy outcomes has not been evaluated in any large-scale study. The Japanese Society of Medical Oncology (JSMO) established its board certification program in 2006 to ensure competency in cancer pharmacotherapy, yet no study has investigated whether this certification translates into measurable differences in patient outcomes.

Guideline-concordant treatment is a well-established quality indicator in oncology, and non-adherence has been associated with inferior survival [7–11]. In colorectal cancer, the complexity of first-line systemic therapy has increased with the incorporation of molecular targeted agents, including anti-VEGF and anti-EGFR antibodies [12]. Optimal regimen selection in metastatic colorectal cancer (mCRC) requires integration of molecular profiling data, assessment of patient fitness, and knowledge of evolving treatment algorithms. Whether the expertise reflected by board certification contributes to more appropriate regimen selection has not been explored.

The SCRUM-Japan MONSTAR-SCREEN observational study is a nationwide genomic screening platform that has enrolled over 10,000 patients with advanced solid tumors across approximately 29 institutions in Japan [13, 14]. The MONSTAR-SCREEN observational study provides a unique opportunity to examine the association between physician certification and treatment outcomes. In this study, the enrolling physician—recorded at the time of informed consent—serves as a proxy for the specialist certification environment of the treating team.

In this study, we used data from the SCRUM-Japan MONSTAR-SCREEN observational study to evaluate the association between the board certification status of the enrolling physician and standard treatment implementation rates across solid tumor types, and to assess whether this certification status was independently associated with overall survival in patients with mCRC.

## Methods

### Study design, data source, and study population

This retrospective cohort study used clinical and genomic data from the SCRUM-Japan MONSTAR-SCREEN observational study, a nationwide, multicenter prospective genomic screening program conducted in Japan. We extracted data from the MONSTAR-SCREEN observational study (UMIN000036749), which enrolled patients between July 2019 and February 2022 at participating institutions across Japan. Key eligibility criteria were: (i) age ≥ 16 years; (ii) written informed consent; (iii) histopathologically confirmed solid tumors with unresectable lesions; (iv) Eastern Cooperative Oncology Group performance status (ECOG PS) of 0 or 1; and (v) life expectancy ≥ 12 weeks at enrollment. For the survival analyses, the study population was restricted to patients with mCRC for three reasons: this group constituted the largest single-cancer cohort in the MONSTAR-SCREEN observational study, providing the statistical power required for multivariable survival analysis; first-line systemic therapy for mCRC is well established among solid tumors, allowing robust covariate adjustment using established prognostic factors (primary tumor sidedness, RAS/BRAF status, and MSI status); and the complexity of regimen selection and patient care in mCRC represents a setting in which specialist expertise is most likely to translate into measurable differences in outcome. The study protocol was approved by the institutional ethics review board of the participating institutions. Data were deidentified prior to analysis to protect patient privacy. Patients were offered the opportunity to opt out in accordance with local regulations. The study was conducted in accordance with the Declaration of Helsinki.

### Exposure and outcome variables

The primary exposure was the JSMO board certification status of the enrolling physician, determined by cross-referencing physician names from informed consent documentation against the JSMO specialist registry as of December 2025. As the enrolling physician may not be the treating physician, this variable was considered a proxy for the specialist environment of the treating team. The primary outcome was overall survival (OS) in patients with mCRC, defined as the time from initiation of first-line systemic therapy to death from any cause. Patients alive at last follow-up were censored. The secondary outcome, assessed across all solid tumors, was the standard treatment implementation rate, defined as the proportion of patients who received a guideline-recommended first-line regimen for the relevant cancer type. Clinical trial participation was counted as guideline-concordant. Guideline concordance for each patient was independently adjudicated by board-certified oncology specialists based on the clinical practice guidelines in effect at the time of treatment initiation (Online Resource 1). Cancer groups were classified as follows: gastrointestinal (esophageal, gastric, small intestinal, and colorectal cancers), hepatobiliary–pancreatic (gallbladder, bile duct, hepatocellular, and pancreatic cancers), urologic (prostate, urothelial, and renal cancers), breast (breast cancer), gynecologic (ovarian, peritoneal, endometrial, and cervical cancers), head and neck (pharyngeal, laryngeal, salivary gland, and thyroid cancers), and other (e.g., sarcoma, skin cancers). For mCRC survival analyses, covariates included age (dichotomized at 65 years), sex, primary tumor sidedness (right vs. left), geographic region (Kanto vs. other), clinical trial participation, use of molecular targeted therapy (bevacizumab, cetuximab, or panitumumab), and number of metastatic organ sites (1, 2–3, or ≥ 4). Tumor molecular profile (RAS mutation, BRAF V600E, and microsatellite instability [MSI] status) was additionally evaluated. Molecular profile data (RAS, BRAF V600E, and MSI status) were obtained from routine clinical diagnostic test results performed at each participating institution and collected through the MONSTAR-SCREEN electronic case report form.

### Statistical analysis

Baseline characteristics were compared using the Mann–Whitney U test for continuous variables and Fisher’s exact test for categorical variables. Standard treatment rates were compared using Fisher’s exact test within cancer groups, geographic regions, and regimen complexity strata. Regimen complexity was defined by the number of drugs administered and classified as combination therapy (≥ 2 drugs) or monotherapy (1 drug); the proportion of patients receiving combination therapy was compared between groups using the chi-square test. The proportion of JSMO board-certified enrolling physicians was calculated for each organ-based cancer group and for each geographic region to characterize the distribution of specialist coverage. At the institutional level, the proportion of board-certified enrolling physicians was determined for each of the 29 participating facilities. The association between institutional specialist proportion and standard treatment implementation rates was examined using a 50% cutoff threshold.

First-line progression-free survival (PFS) in mCRC was defined as the time from initiation of first-line systemic therapy to disease progression or death from any cause, whichever occurred first. Patients without a documented progression or death event were censored at the date of last treatment or follow-up. PFS was estimated using the Kaplan–Meier method and compared between certification groups using the log-rank test; this analysis was exploratory and no formal multiplicity adjustment was applied. OS was estimated using the Kaplan–Meier method and compared using the log-rank test. Hazard ratios (HRs) with 95% confidence intervals (CIs) were estimated using Cox proportional hazards regression. Sequential models were fitted: a univariate model, Model 1 adjusting for age, sex, primary tumor sidedness, region, and trial participation; Model 2 additionally adjusting for molecular targeted therapy; and Model 3 further adjusting for metastatic organ count. Two additional models were constructed to assess the robustness of the certification effect against tumor molecular profile: Model 4a additionally adjusted for RAS and BRAF V600E mutation status, and Model 4b further adjusted for MSI status. To quantify robustness against unmeasured confounding, we performed E-value sensitivity analysis [15]. Pre-specified subgroup analyses were performed, and a multiplicative interaction term tested regimen complexity as an effect modifier. All analyses used R version 4.3 (R Foundation for Statistical Computing). Two-sided *p* < 0.05 denoted statistical significance.

## Results

### Patient characteristics

**Fig. 1:**
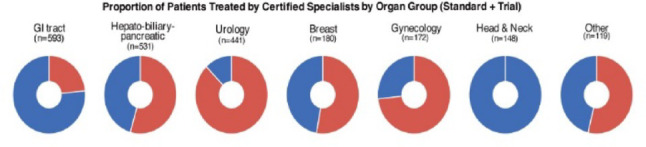
Proportion of patients enrolled by certified specialists by organ group. Each pie chart represents the percentage of patients managed by Japanese Society of Medical Oncology (JSMO) board-certified specialists within each organ group. Sample sizes are indicated in parentheses

A total of 2,184 patients with advanced solid tumors who received first-line systemic therapy were included. Of these, 1,084 (49.6%) were enrolled by JSMO board-certified physicians and 1,100 (50.4%) by non–board-certified physicians. Clinical trial participants (*n* = 106) were included, with trial participation counted as guideline-concordant treatment. The board-certified group had a significantly lower proportion of patients aged ≥ 65 years (53.8% vs. 61.7%; *p* < 0.001). Cancer type distribution differed substantially between groups (*p* < 0.001). Gastrointestinal cancers predominated in the board-certified group (41.9%), whereas urologic cancers were most frequent in the non–board-certified group (35.2%). All head and neck cancer patients (*n* = 148) were enrolled by board-certified physicians (Fig. 1). Geographic distribution also differed significantly (*p* < 0.001): the proportion enrolled by board-certified physicians ranged from 5.0% in Kyushu/Okinawa to 88.7% in Chubu. Clinical trial participation was significantly more frequent in the non–board-certified group (5.8% vs. 3.7%; *p* = 0.025, Table 1).

**Table 1:** Baseline patient characteristics

Characteristic	Board-certified (*n* = 1,084)	Non–board-certified (*n* = 1,100)	*p* value^a^
Age ≥ 65 years, n (%)	583 (53.8)	679 (61.7)	< 0.001
Male sex, n (%)	597 (55.1)	612 (55.6)	0.825
Cancer type, n (%)			< 0.001
Gastrointestinal	454 (41.9)	139 (12.6)	
Hepatobiliary-pancreatic	242 (22.3)	289 (26.3)	
Urological	54 (5.0)	387 (35.2)	
Gynecological	46 (4.2)	126 (11.5)	
Head and neck	148 (13.7)	0 (0.0)	
Breast	85 (7.8)	95 (8.6)	
Other	55 (5.1)	64 (5.8)	
Geographic region, n (%)			< 0.001
Hokkaido/Tohoku	29 (2.7)	59 (5.4)	
Kanto	545 (50.3)	664 (60.4)	
Chubu	289 (26.7)	37 (3.4)	
Kinki	170 (15.7)	110 (10.0)	
Chugoku/Shikoku	40 (3.7)	21 (1.9)	
Kyushu/Okinawa	11 (1.0)	209 (19.0)	
Clinical trial participation, n (%)	40 (3.7)	64 (5.8)	0.025
Standard treatment (trial-inclusive)^b^, n (%)	1052 (97.0)	1066 (96.9)	0.884
Combination therapy (≥ 2 drugs), n (%)	879 (81.1)	887 (80.6)	0.830
Median follow-up, months	22.2	28.5	< 0.001

The enrolling physician (recorded at informed consent) serves as a proxy for the specialist certification environment of the treating team

^a^Median follow-up was compared using the Mann–Whitney U test; categorical variables were compared using the chi-square test or Fisher’s exact test

^b^Standard treatment (trial-inclusive): guideline-recommended first-line regimen or clinical trial participation

*p < 0.05; **p < 0.01; ***p < 0.001

### Standard treatment implementation rates

**Fig. 2:**
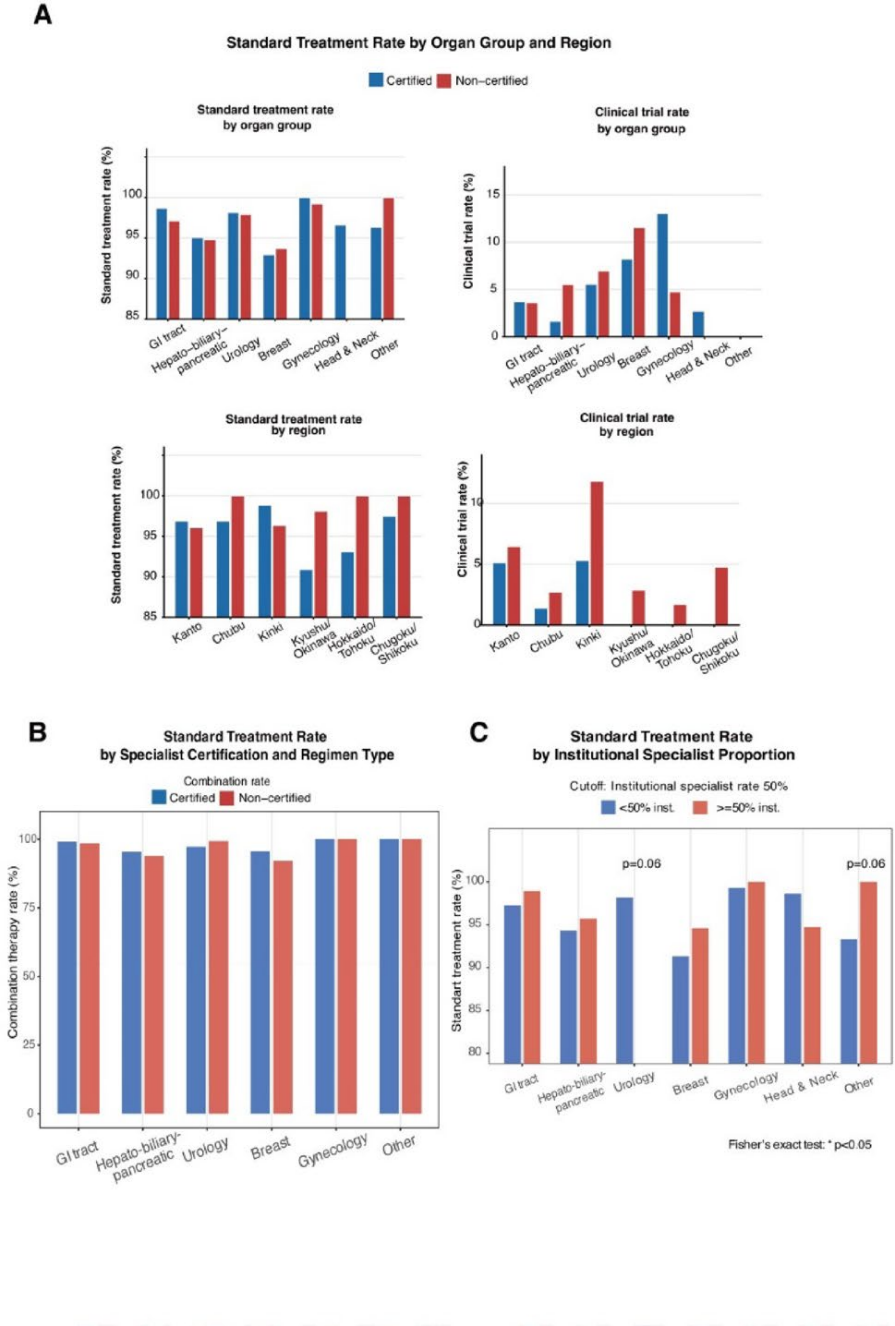
Standard treatment rates, combination therapy rates, and institutional specialist density by cancer type and geographic region. **A** Standard treatment rates (left), non-standard treatment rates (middle), and clinical trial participation rates (right) by cancer type (upper panels) and geographic region (lower panels), stratified by board certification status of the enrolling physician. Clinical trial participation was counted as standard treatment. **B** Combination therapy rates (≥ 2 drugs) by cancer type, stratified by board certification status. **C** Standard treatment rates by cancer type, stratified by institutional specialist density (cutoff: ≥50% vs. <50% board-certified physicians per institution). Dashed line indicates 100%. p-values shown where *p* < 0.10

The overall standard treatment implementation rate was uniformly high (board-certified, 97.0%; non–board-certified, 96.9%). When stratified by organ group and geographic region, no significant differences were observed between the two groups (Fig. 2A). Similarly, no significant difference was observed when stratified by regimen complexity: combination therapy (97.4% vs. 97.2%; OR, 1.08; *p* = 0.884, Fig. 2B). At the institutional level (29 facilities), no significant association was found between the proportion of board-certified physicians and standard treatment rates (Fig. 2C).

### Survival and subgroup analysis in mCRC

**Fig. 3:**
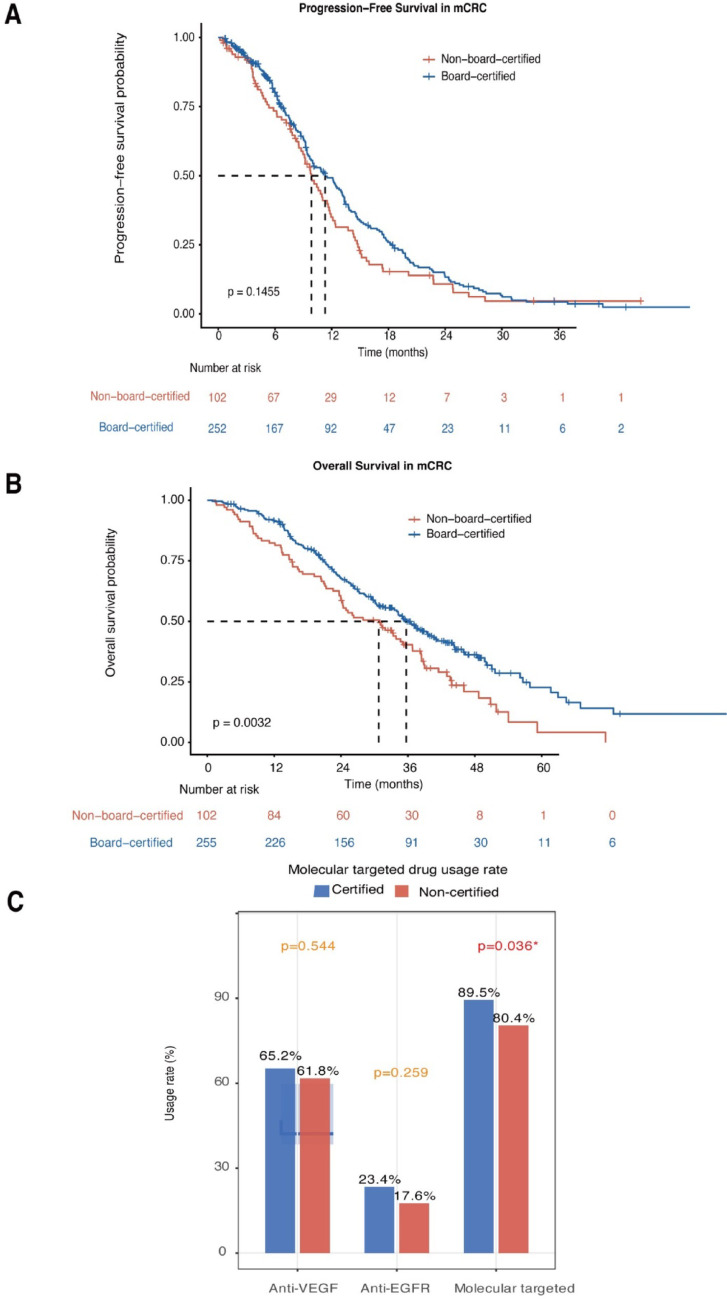
Progression-free survival, overall survival, and molecular targeted drug usage in patients with metastatic colorectal cancer receiving first-line treatment, stratified by specialist certification status. **(A)** Kaplan–Meier curves for first-line progression-free survival comparing board-certified (*n* = 252) and non–board-certified (*n* = 102) physician groups (log-rank test, *p* = 0.145). **(B)** Kaplan–Meier curves for overall survival comparing board-certified (*n* = 256) and non–board-certified (*n* = 102) physician groups (log-rank test, *p* = 0.003). **(C)** Usage rates of anti-VEGF agents, anti-EGFR agents, and any molecular targeted therapy in board-certified versus non–board-certified groups. Statistical significance was assessed using Fisher’s exact test (*p < 0.05)

**Fig. 4:**
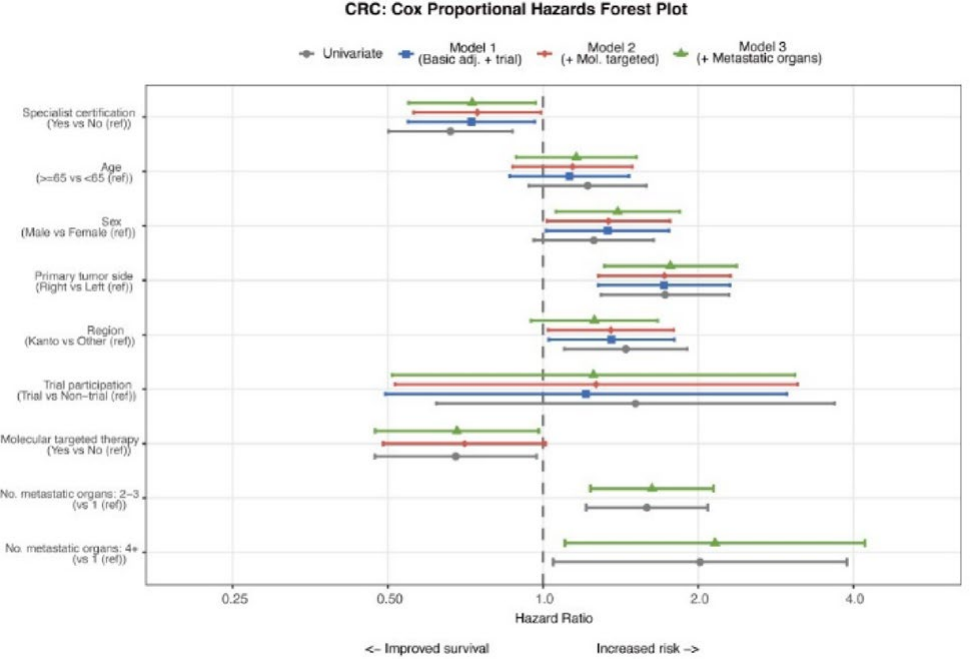
Forest plot of Cox proportional hazards regression analysis for overall survival in patients with metastatic colorectal cancer. Hazard ratios and 95% confidence intervals are shown for univariate analysis and three nested multivariate models: Model 1 (specialist certification adjusted for age, sex, primary tumor sidedness, geographic region, and trial participation); Model 2 (Model 1 plus molecular targeted therapy use); Model 3 (Model 2 plus number of metastatic organs [categorized as 1 (reference), 2–3, and 4+]). Hazard ratios < 1.0 indicate improved survival

Among 358 patients with mCRC, 256 (71.5%) were enrolled by board-certified physicians and 102 (28.5%) by non–board-certified physicians. For the PFS analysis, four patients with missing progression data were excluded, yielding an evaluable population of 354 (board-certified, *n* = 252; non–board-certified, *n* = 102). Median PFS was 11.3 months (95% CI 9.7–13.2) in the board-certified group and 9.9 months (95% CI 8.8–11.7) in the non–board-certified group. On univariate analysis, board certification was associated with a numerically lower hazard of progression or death, though this did not reach statistical significance (HR, 0.82; 95% CI 0.63–1.07; log-rank *p* = 0.145; Fig. 3A). When stratified by drug class, anti-VEGF agent use was numerically higher in the board-certified group (65.2% vs. 61.8%; *p* = 0.544), as was anti-EGFR agent use (23.4% vs. 17.6%; *p* = 0.259), though neither difference reached statistical significance. OS was analyzed in all 358 patients with mCRC. The board-certified group had significantly longer OS (median, 36.5 vs. 30.7 months; log-rank *p* = 0.003; Fig. 3B). The 12-month PFS rates were 49.2% (95% CI 42.8–56.6%) and 35.0% (95% CI 26.2–46.6%), respectively. Board-certified physicians used any molecular targeted therapy more frequently than non–board-certified physicians (89.5% vs. 80.4%; *p* = 0.036; Fig. 3C). In univariate Cox regression, board certification was associated with a 34% reduction in the hazard of death (HR, 0.66; 95% CI 0.50–0.87; *p* = 0.003; Table 2). After sequential adjustment, the association remained significant: Model 1 (HR, 0.73; 95% CI 0.55–0.96; *p* = 0.027), Model 2 (HR, 0.75; 95% CI 0.56–0.99; *p* = 0.043), and Model 3 (HR, 0.73; 95% CI 0.55–0.97; *p* = 0.029). Right-sided primary tumors (HR, 1.77; *p* < 0.001), male sex (HR, 1.40; *p* = 0.018), and higher metastatic organ count (2–3 organs: HR, 1.63; ≥4 organs: HR, 2.16) were also associated with inferior OS in the fully adjusted model (Fig. 4; Table 2). The association between board certification and improved OS was consistent across pre-specified subgroups. The survival benefit was significant among patients aged ≥ 65 years (HR, 0.56; 95% CI 0.38–0.83; *p* = 0.004), males (HR, 0.64; 95% CI 0.45–0.91; *p* = 0.014), and recipients of molecular targeted therapy (HR, 0.69; 95% CI 0.51–0.94; *p* = 0.020). Among patients aged < 65 years, the point estimate favored the board-certified group but was not statistically significant (HR, 0.76; 95% CI 0.51–1.14; *p* = 0.186, Table 3). After additional adjustment for tumor molecular profile, the HR for board certification was 0.64 (95% CI 0.47–0.88; *p* = 0.005) in Model 4a (*n* = 323; adjusted for RAS and BRAF V600E) and 0.59 (95% CI 0.40–0.85; *p* = 0.005) in Model 4b (*n* = 235; additionally adjusted for MSI) (Supplementary Figure S, Online Resource 2). The between-group distributions of RAS, BRAF V600E, and MSI were comparable (40.2% vs. 39.2%, *p* = 0.903; 16.2% vs. 13.0%, *p* = 0.608; 4.4% vs. 4.2%, *p* = 1.000, respectively). The E-values were 2.04 for Model 3 (lower 95% CI bound, 1.14) and 2.48 for Model 4a (lower 95% CI bound, 1.52) (Online Resource 3).

**Table 2:** Cox proportional hazards regression for overall survival in metastatic colorectal cancer *n* = 357; 226 events

Variable	HR^a^	95% CI^a^	*p* value
Univariate			
Specialist certification (Yes vs. No [ref])	0.66	0.50–0.87	0.003**
Age (≥ 65 vs. < 65 [ref])	1.22	0.94–1.59	0.136
Sex (Male vs. Female [ref])	1.26	0.96–1.64	0.096
Primary tumor side (Right vs. Left [ref])	1.73	1.30–2.30	< 0.001***
Region (Kanto vs. Other [ref])	1.45	1.10–1.91	0.008**
Trial participation (Trial vs. Non-trial [ref])	1.51	0.62–3.68	0.362
Molecular targeted therapy (Yes vs. No [ref])	0.68	0.47–0.97	0.034*
No. metastatic organs: 2–3 (vs. 1 [ref])	1.59	1.21–2.09	< 0.001***
No. metastatic organs: ≥4 (vs. 1 [ref])	2.02	1.05–3.89	0.036*
Model 1^b^			
Specialist certification (Yes vs. No [ref])	0.73	0.55–0.96	0.027*
Age (≥ 65 vs. < 65 [ref])	1.13	0.86–1.47	0.387
Sex (Male vs. Female [ref])	1.34	1.01–1.76	0.039*
Primary tumor side (Right vs. Left [ref])	1.72	1.28–2.31	< 0.001***
Region (Kanto vs. Other [ref])	1.36	1.03–1.80	0.032*
Trial participation (Trial vs. Non-trial [ref])	1.21	0.49–2.97	0.675
Model 2^c^			
Specialist certification (Yes vs. No [ref])	0.75	0.56–0.99	0.043*
Age (≥ 65 vs. < 65 [ref])	1.14	0.87–1.49	0.333
Sex (Male vs. Female [ref])	1.34	1.02–1.76	0.037*
Primary tumor side (Right vs. Left [ref])	1.72	1.28–2.31	< 0.001***
Region (Kanto vs. Other [ref])	1.35	1.02–1.79	0.034*
Trial participation (Trial vs. Non-trial [ref])	1.27	0.52–3.12	0.604
Molecular targeted therapy (Yes vs. No [ref])	0.70	0.49–1.01	0.059
Model 3^d^			
Specialist certification (Yes vs. No [ref])	0.73	0.55–0.97	0.029*
Age (≥ 65 vs. < 65 [ref])	1.16	0.89–1.52	0.276
Sex (Male vs. Female [ref])	1.40	1.06–1.84	0.018*
Primary tumor side (Right vs. Left [ref])	1.77	1.31–2.37	< 0.001***
Region (Kanto vs. Other [ref])	1.26	0.95–1.67	0.111
Trial participation (Trial vs. Non-trial [ref])	1.25	0.51–3.08	0.623
Molecular targeted therapy (Yes vs. No [ref])	0.68	0.47–0.98	0.040*
No. metastatic organs: 2–3 (vs. 1 [ref])	1.63	1.24–2.14	< 0.001***
No. metastatic organs: ≥4 (vs. 1 [ref])	2.16	1.10–4.21	0.025*

^a^ HR, hazard ratio; CI, confidence interval

^b^ Model 1: adjusted for age (≥ 65 vs. < 65), sex, primary tumor sidedness (right vs. left), region (Kanto vs. other), and clinical trial participation

^c^ Model 2: Model 1 + molecular targeted therapy (bevacizumab, cetuximab, or panitumumab)

^d^ Model 3: Model 2 + number of metastatic organ sites (1 [ref], 2–3, ≥ 4)

*p < 0.05; **p < 0.01; ***p < 0.001

**Table 3:** Subgroup analysis: association between board certification and overall survival in metastatic colorectal cancer

Subgroup	*n*	Events	HR^a^	95% CI^a^	*p* value	*p* interaction^b^
All mCRC	358	226	0.66	0.50–0.87	0.003**	
Age						0.249
< 65 years	191	120	0.76	0.51–1.14	0.186	
≥ 65 years	167	106	0.56	0.38–0.83	0.004**	
Sex						0.787
Male	203	135	0.64	0.45–0.91	0.014*	
Female	155	91	0.69	0.45–1.07	0.101	
Primary tumor side						0.270
Left	259	156	0.61	0.44–0.86	0.004**	
Right	98	70	0.85	0.51–1.39	0.508	
Region						0.037
Kanto	114	79	0.52	0.33–0.82	0.005**	
Other	244	147	0.87	0.60–1.27	0.474	
Molecular targeted therapy						0.597
Yes	311	190	0.69	0.51–0.94	0.020*	
No	47	36	0.59	0.30–1.15	0.120	

^a^ HR, hazard ratio; CI, confidence interval. Univariate Cox regression for specialist certification (Yes vs. No [ref]) within each subgroup

^b^ p interaction: multiplicative interaction term between certification and the subgroup variable

Monotherapy subgroup: insufficient sample size for analysis (*n* = 9)

mCRC, metastatic colorectal cancer

*p < 0.05; **p < 0.01; ***p < 0.001

## Discussion

In this nationwide cohort study using the SCRUM-Japan MONSTAR-SCREEN observational study, enrollment by a JSMO board-certified medical oncologist was independently associated with a 27–34% reduction in the hazard of death among patients with mCRC, an association that persisted across sequential multivariable models. This survival advantage was observed despite virtually identical standard treatment implementation rates (97.0% vs. 96.9%), suggesting that the benefit of specialist involvement extends beyond guideline adherence.

The near-equivalence of guideline-concordant treatment rates is itself noteworthy. This uniformly high adherence likely reflects the academic nature of the SCRUM-Japan participating institutions, where both certified and non-certified physicians practice within infrastructure that supports guideline-concordant care. That OS differed despite comparable treatment selection implies that specialist care encompasses dimensions not captured by binary measures of guideline adherence—potentially including dose optimization, adverse event management, treatment modification timing, and supportive care integration [16, 17].

Our results suggest that differential use of molecular targeted therapy may partly account for the specialist survival advantage. Board-certified physicians used molecular targeted therapy more frequently (89.5% vs. 80.4%; *p* = 0.036), and the HR was attenuated from Model 1 (0.73) to Model 2 (0.75) after adjustment for this variable. However, the association remained significant in Model 3 (HR, 0.73; *p* = 0.029), indicating that molecular targeted therapy use explains only part of the survival benefit. Unmeasured aspects—such as anti-VEGF versus anti-EGFR agent selection based on sidedness and molecular profiling, dose adjustments, and sequencing of subsequent therapies—may also contribute [18]. Although first-line PFS did not reach statistical significance (HR, 0.82; 95% CI 0.63–1.07; *p* = 0.145), the directionally consistent trend suggests a modest benefit in tumor control that may partly reflect this higher use of molecular targeted therapy. The more pronounced OS benefit relative to the attenuated PFS effect implies that factors beyond first-line tumor control also contribute.

The subgroup analyses further characterize the population in which specialist certification confers the greatest benefit. The survival advantage was most pronounced in patients aged ≥ 65 years (HR, 0.56; *p* = 0.004), male patients (HR, 0.64; *p* = 0.014), and those with left-sided primary tumors (HR, 0.61; *p* = 0.004), although no statistically significant interactions were observed for molecular targeted therapy use. These findings suggest that the benefit of board-certified oncologists extends beyond the selection of specific agents and likely encompasses broader aspects of treatment management, including dose optimization, toxicity monitoring, and supportive care coordination.

The distribution of board-certified physicians across cancer types reflects the historical development of subspecialty practice in Japan. Cancers traditionally managed by surgical subspecialties—such as urologic and gynecologic malignancies—had substantially lower proportions of JSMO-certified enrolling physicians, whereas head and neck cancers were exclusively enrolled by board-certified physicians. Despite this marked heterogeneity in specialist coverage, standard treatment implementation rates were uniformly high across all cancer groups and geographic regions. This finding suggests that institutional infrastructure, including multidisciplinary tumor boards, may be sufficient to maintain the standard treatment implementation rates regardless of specialist certification status. It also implies that the quality gap attributable to physician certification may be more evident in aspects of care that lie beyond binary guideline adherence, such as regimen optimization, greater continuity of care, higher rates of subsequent-line therapy, and more appropriate patient selection for complex therapies or later-line settings.

From a health policy perspective, these results support the continued development of JSMO board certification. The JSMO system was established to ensure competency in cross-organ medical oncology, recognizing that complex systemic therapies demand dedicated expertise [19]. Our finding that board-certified physicians achieved better survival in mCRC provides empirical evidence for the clinical value of this training pathway. The proportion of patients treated by a JSMO board-certified oncologist varied markedly across institutions and regions, ranging from 5.0% in Kyushu/Okinawa to 88.7% in Chubu. This variation likely reflects differences in the certification status of treating physicians at participating institutions rather than regional specialist density, and warrants further investigation into equitable access to board-certified oncologists.

Several limitations should be acknowledged. First, SCRUM-Japan comprises institutions with high academic activity and commitment to genomic screening. Even non-certified physicians in this cohort practiced within highly specialized environments with multidisciplinary tumor boards and clinical trial infrastructure. This may have attenuated the detectable difference between groups, suggesting that our estimate is conservative compared with what might be observed in community settings [9, 10]. Second, the JSMO certification status of the enrolling physician was used as a proxy for the specialist environment of the treating team, and the enrolling physician may not have been the primary treating physician throughout the disease course. In addition, certification status was ascertained from the JSMO registry as of December 2025, whereas treatment occurred between 2019 and 2022. These sources of exposure misclassification were likely non-differential with respect to survival and would therefore be expected to attenuate the observed association toward the null. Nevertheless, the magnitude of this misclassification could not be directly quantified. Third, although the MONSTAR-SCREEN observational study restricted enrollment to patients with ECOG PS 0 or 1, PS itself was not collected, and residual confounding by PS variability, comorbidity burden, socioeconomic status, and institutional resources cannot be excluded. The E-value of 2.48 indicates that an unmeasured confounder would need to be associated with both certification and OS by a hazard ratio of at least 2.48 to nullify the observed association; such a magnitude is unlikely to be reached by residual factors within a cohort restricted to PS 0–1. Board certification status was assessed at first-line enrollment, whereas OS reflects cumulative effects across all treatment lines; disentangling first-line decision-making from subsequent management was not possible. The survival analysis was furthermore restricted to mCRC, and detailed longitudinal treatment data, including dose intensity, subsequent therapies, and supportive care, were unavailable, preventing detailed mechanistic analysis. Whether the specialist survival advantage extends to cancer types other than mCRC remains to be determined, as larger disease-specific cohorts would be required to draw definitive conclusions. Our survival findings are mCRC-specific and should not be extrapolated to other cancer types.

In this nationwide cohort study, enrollment by a JSMO board-certified medical oncologist was independently associated with improved OS in mCRC, despite comparable guideline adherence. The survival benefit was partially mediated by higher molecular targeted therapy utilization but not fully explained by this factor alone. These findings provide real-world evidence supporting the clinical value of medical oncologist certification and highlight the need for investment in specialist training, equitable regional distribution, and prospective studies to elucidate the specific mechanisms through which specialist care improves survival.

## Supplementary Information

Below is the link to the electronic supplementary material.

Supplementary Material 1
