# Supplementary material for: Impact of medical oncologist certification on survival outcomes in metastatic colorectal cancer: evidence from the SCRUM-Japan MONSTAR-SCREEN Observational Study
Source: Int J Clin Oncol. 2026 Jun 6;31(8):1431–40. doi: 10.1007/s10147-026-03069-0 (PMC13401523; doi:10.1007/s10147-026-03069-0)
Supplement: Supplementary file 1 — Supplementary Material 1 [file 10147_2026_3069_MOESM1_ESM.docx]

**Online Resource 1. Cancer type, histology, and regimen pairs with standard therapy classification**

| **Cancer type^a^** | **Histology** | **Regimen** | **Standard therapy^b^** |
| --- | --- | --- | --- |
| AC | Adenocarcinoma | 5-FU,Bevacizumab,Irinotecan,Oxaliplatin | Yes |
| AC | NoData | Bevacizumab,Capecitabine,Oxaliplatin | Yes |
| BC | Apocrine Carcinoma | Docetaxel,Pertuzumab,Trastuzumab | Yes |
| BC | Invasive Lobular Carcinoma | Abemaciclib,Letrozole | Yes |
| BC | Invasive Lobular Carcinoma | Anastrozole | Yes |
| BC | Invasive Lobular Carcinoma | Abemaciclib,Anastrozole | Yes |
| BC | Invasive Lobular Carcinoma | Abemaciclib,Fulvestrant | Yes |
| BC | Invasive Lobular Carcinoma | Atezolizumab,Nab-Paclitaxel | Yes |
| BC | Invasive Lobular Carcinoma | Docetaxel | Yes |
| BC | Invasive Lobular Carcinoma | Leuprorelin,Tamoxifen | Yes |
| BC | Invasive Lobular Carcinoma | Nab-Paclitaxel | Yes |
| BC | Invasive Micropapillary Carcinoma | Abemaciclib,Letrozole | Yes |
| BC | Invasive Micropapillary Carcinoma | Docetaxel,Pertuzumab,Trastuzumab | Yes |
| BC | Invasive ductal carcinoma NOS | Abemaciclib,Fulvestrant | Yes |
| BC | Invasive ductal carcinoma NOS | Abemaciclib,Letrozole | Yes |
| BC | Invasive ductal carcinoma NOS | Docetaxel,Pertuzumab,Trastuzumab | Yes |
| BC | Invasive ductal carcinoma NOS | Abemaciclib,Anastrozole | Yes |
| BC | Invasive ductal carcinoma NOS | Abemaciclib,Fulvestrant,Goserelin | Yes |
| BC | Invasive ductal carcinoma NOS | Leuprorelin,Tamoxifen | Yes |
| BC | Invasive ductal carcinoma NOS | Atezolizumab,Nab-Paclitaxel | Yes |
| BC | Invasive ductal carcinoma NOS | Tamoxifen | Yes |
| BC | Invasive ductal carcinoma NOS | Anastrozole | Yes |
| BC | Invasive ductal carcinoma NOS | Cyclophosphamide,Doxorubicin | Yes |
| BC | Invasive ductal carcinoma NOS | Letrozole | Yes |
| BC | Invasive ductal carcinoma NOS | Trastuzumab Emtansine (T-DM1) | Yes |
| BC | Invasive ductal carcinoma NOS | 5-FU,Cyclophosphamide,Docetaxel,Epirubicin | Yes |
| BC | Invasive ductal carcinoma NOS | Abemaciclib,Denosumab,Letrozole | Yes |
| BC | Invasive ductal carcinoma NOS | Anastrozole,Paclitaxel | Yes |
| BC | Invasive ductal carcinoma NOS | Bevacizumab,Paclitaxel | Yes |
| BC | Invasive ductal carcinoma NOS | Capecitabine,Trastuzumab | No |
| BC | Invasive ductal carcinoma NOS | Cyclophosphamide,Epirubicin | Yes |
| BC | Invasive ductal carcinoma NOS | Cyclophosphamide,Pirarubicin | No |
| BC | Invasive ductal carcinoma NOS | Eribulin,Pertuzumab,Trastuzumab | Yes |
| BC | Invasive ductal carcinoma NOS | Everolimus,Exemestane | No |
| BC | Invasive ductal carcinoma NOS | Everolimus,Letrozole | No |
| BC | Invasive ductal carcinoma NOS | Exemestane | Yes |
| BC | Invasive ductal carcinoma NOS | Fulvestrant | Yes |
| BC | Invasive ductal carcinoma NOS | Goserelin,Letrozole | Yes |
| BC | Invasive ductal carcinoma NOS | Goserelin,Tamoxifen | Yes |
| BC | Invasive ductal carcinoma NOS | Letrozole,Leuprorelin | Yes |
| BC | Invasive ductal carcinoma NOS | Medroxyprogesterone Acetate | No |
| BC | Invasive ductal carcinoma NOS | Paclitaxel,Pertuzumab,Trastuzumab | Yes |
| BC | Invasive ductal carcinoma NOS | Paclitaxel,Trastuzumab | No |
| BC | Invasive ductal carcinoma NOS | Pertuzumab,Tamoxifen,Trastuzumab | Yes |
| BC | Invasive ductal carcinoma NOS | Trastuzumab | No |
| BC | Medullary Carcinoma | Pertuzumab,Trastuzumab,Vinorelbine | No |
| BC | Mucinous Carcinoma | 5-FU,Cyclophosphamide,Epirubicin | Yes |
| BC | Mucinous Carcinoma | Abemaciclib,Fulvestrant,Goserelin | Yes |
| BC | Other | Goserelin,Tamoxifen | Yes |
| BC | Other | Letrozole | Yes |
| BC | Scirrhous Type | Abemaciclib,Fulvestrant | Yes |
| BC | Scirrhous Type | Atezolizumab,Nab-Paclitaxel | Yes |
| BC | Scirrhous Type | Docetaxel,Pertuzumab,Trastuzumab | Yes |
| BC | Scirrhous Type | Abemaciclib,Fulvestrant,Goserelin | Yes |
| BC | Scirrhous Type | Abemaciclib,Anastrozole | Yes |
| BC | Scirrhous Type | Abemaciclib,Letrozole | Yes |
| BC | Scirrhous Type | Anastrozole,Goserelin | Yes |
| BC | Scirrhous Type | Trastuzumab Emtansine (T-DM1) | Yes |
| BC | Scirrhous Type | Abemaciclib,Anastrozole,Goserelin,Letrozole | Yes |
| BC | Scirrhous Type | Abemaciclib,Exemestane | Yes |
| BC | Scirrhous Type | Abemaciclib,Exemestane,Goserelin | Yes |
| BC | Scirrhous Type | Abemaciclib,Fulvestrant,Goserelin,Palbociclib | Yes |
| BC | Scirrhous Type | Abemaciclib,Fulvestrant,Leuprorelin | Yes |
| BC | Scirrhous Type | Abemaciclib,Letrozole,Leuprorelin | Yes |
| BC | Scirrhous Type | Anastrozole | Yes |
| BC | Scirrhous Type | Capecitabine | Yes |
| BC | Scirrhous Type | Capecitabine,Trastuzumab | No |
| BC | Scirrhous Type | Docetaxel | Yes |
| BC | Scirrhous Type | Docetaxel,Goserelin,Pertuzumab,Trastuzumab | Yes |
| BC | Scirrhous Type | Doxorubicin,Pertuzumab,Trastuzumab | No |
| BC | Scirrhous Type | Eribulin,Pertuzumab,Trastuzumab | Yes |
| BC | Scirrhous Type | Fulvestrant,Goserelin | Yes |
| BC | Scirrhous Type | Fulvestrant,Goserelin,Palbociclib | Yes |
| BC | Scirrhous Type | Goserelin,Tamoxifen | Yes |
| BC | Scirrhous Type | Letrozole,Leuprorelin | Yes |
| BC | Scirrhous Type | Nab-Paclitaxel,Pembrolizumab,Trastuzumab | Yes |
| BC | Scirrhous Type | Paclitaxel | Yes |
| BC | Scirrhous Type | Paclitaxel,Pertuzumab,Trastuzumab | Yes |
| BC | Scirrhous Type | Pertuzumab,Trastuzumab,Vinorelbine | No |
| BC | Scirrhous Type | S-1 | Yes |
| BC | Scirrhous Type | Tamoxifen | Yes |
| BC | Scirrhous Type | Trastuzumab | No |
| BC | Solid Type | Abemaciclib,Fulvestrant | Yes |
| BC | Solid Type | Capecitabine | Yes |
| BC | Solid Type | Abemaciclib,Fulvestrant,Goserelin | Yes |
| BC | Solid Type | Abemaciclib,Fulvestrant,Leuprorelin | Yes |
| BC | Solid Type | Abemaciclib,Letrozole | Yes |
| BC | Solid Type | Atezolizumab,Nab-Paclitaxel | Yes |
| BC | Solid Type | Bevacizumab,Paclitaxel | Yes |
| BC | Solid Type | Goserelin,Tamoxifen | Yes |
| BC | Solid Type | Trastuzumab Deruxtecan | Yes |
| BC | Tubule Forming Type | 5-FU,Cyclophosphamide,Epirubicin | Yes |
| BC | Tubule Forming Type | Abemaciclib,Anastrozole,Goserelin | Yes |
| BC | Tubule Forming Type | Docetaxel,Pertuzumab,Trastuzumab | Yes |
| BC | Tubule Forming Type | Goserelin,Tamoxifen | Yes |
| BTC | Adenocarcinoma | Cisplatin,Gemcitabine | Yes |
| BTC | Adenocarcinoma | Cisplatin,Gemcitabine,S-1 | Yes |
| BTC | Adenocarcinoma | Gemcitabine,S-1 | Yes |
| BTC | Adenocarcinoma | Gemcitabine | Yes |
| BTC | Adenocarcinoma | 5-FU,Irinotecan,Levofolinate,Oxaliplatin | No |
| BTC | Adenocarcinoma | Cisplatin | No |
| BTC | Combined Hepatocellular And Cholangiocarcinoma | Cisplatin,Gemcitabine | Yes |
| BTC | Other | Cisplatin,Gemcitabine | Yes |
| BTC | Other | Cisplatin,Gemcitabine,S-1 | Yes |
| BTC | Squamous Cell Carcinoma | Cisplatin,Gemcitabine | Yes |
| CESC | Adenocarcinoma | Carboplatin,Paclitaxel | Yes |
| CESC | Endometrioid Carcinoma | Bevacizumab,Cisplatin,Paclitaxel | Yes |
| CESC | Mucinous Carcinoma | Bevacizumab,Carboplatin,Paclitaxel | Yes |
| CESC | Mucinous Carcinoma | Cisplatin | Yes |
| CESC | Mucinous Carcinoma, Gastric Type | Bevacizumab,Carboplatin,Paclitaxel | Yes |
| CESC | Mucinous Carcinoma, Gastric Type | Carboplatin,Paclitaxel | Yes |
| CESC | Squamous Cell Carcinoma | Bevacizumab,Carboplatin,Paclitaxel | Yes |
| CESC | Squamous Cell Carcinoma | Cisplatin | Yes |
| CESC | Squamous Cell Carcinoma | Bevacizumab,Cisplatin,Paclitaxel | Yes |
| CESC | Squamous Cell Carcinoma | Carboplatin,Paclitaxel | Yes |
| CESC | Squamous Cell Carcinoma, Keratinizing Type | Cisplatin | Yes |
| CESC | Squamous Cell Carcinoma, Non-Keratinizing Type | Bevacizumab,Carboplatin,Paclitaxel | Yes |
| CESC | Squamous Cell Carcinoma, Non-Keratinizing Type | Carboplatin,Paclitaxel | Yes |
| CESC | Squamous Cell Carcinoma, Non-Keratinizing Type | Cisplatin | Yes |
| CESC | Undifferentiated Carcinoma | Carboplatin,Paclitaxel | Yes |
| CESC | Undifferentiated Carcinoma | Cisplatin | Yes |
| CRC | Adenocarcinoma | Bevacizumab,Capecitabine,Oxaliplatin | Yes |
| CRC | Adenocarcinoma | 5-FU,Oxaliplatin,Panitumumab | Yes |
| CRC | Adenocarcinoma | 5-FU,Bevacizumab,Oxaliplatin | Yes |
| CRC | Adenocarcinoma | 5-FU,Bevacizumab,Irinotecan,Oxaliplatin | Yes |
| CRC | Adenocarcinoma | 5-FU,Bevacizumab,Levofolinate,Oxaliplatin | Yes |
| CRC | Adenocarcinoma | Capecitabine,Oxaliplatin | Yes |
| CRC | Adenocarcinoma | Bevacizumab,Capecitabine,Irinotecan,Oxaliplatin | Yes |
| CRC | Adenocarcinoma | 5-FU,Bevacizumab,Irinotecan,Levofolinate,Oxaliplatin | Yes |
| CRC | Adenocarcinoma | 5-FU,Levofolinate,Oxaliplatin | Yes |
| CRC | Adenocarcinoma | 5-FU,Levofolinate,Oxaliplatin,Panitumumab | Yes |
| CRC | Adenocarcinoma | 5-FU,Bevacizumab,Irinotecan | Yes |
| CRC | Adenocarcinoma | Bevacizumab,Oxaliplatin,S-1 | Yes |
| CRC | Adenocarcinoma | Bevacizumab,Irinotecan,S-1 | Yes |
| CRC | Adenocarcinoma | 5-FU,Cetuximab,Oxaliplatin | Yes |
| CRC | Adenocarcinoma | 5-FU,Oxaliplatin | Yes |
| CRC | Adenocarcinoma | 5-FU,Cetuximab,Irinotecan | Yes |
| CRC | Adenocarcinoma | Bevacizumab,Capecitabine | Yes |
| CRC | Adenocarcinoma | 5-FU,Irinotecan,Oxaliplatin | Yes |
| CRC | Adenocarcinoma | 5-FU,Arfolitixorin,Bevacizumab,Oxaliplatin | Trial |
| CRC | Adenocarcinoma | 5-FU,Irinotecan,Panitumumab | Yes |
| CRC | Adenocarcinoma | 5-FU,Irinotecan,Ramucirumab | Yes |
| CRC | Adenocarcinoma | Bevacizumab,S-1 | Yes |
| CRC | Adenocarcinoma | Capecitabine | Yes |
| CRC | Adenocarcinoma | Panitumumab | Yes |
| CRC | Adenocarcinoma | Pembrolizumab | Yes |
| CRC | Adenocarcinoma | UFT | No |
| CRC | Adenocarcinoma | 5-FU,Bevacizumab,Capecitabine,Irinotecan,Oxaliplatin | Yes |
| CRC | Adenocarcinoma | 5-FU,Bevacizumab,Capecitabine,Oxaliplatin | Yes |
| CRC | Adenocarcinoma | 5-FU,Bevacizumab,Irinotecan,Levofolinate | Yes |
| CRC | Adenocarcinoma | 5-FU,Cetuximab,Irinotecan,Oxaliplatin | Yes |
| CRC | Adenocarcinoma | 5-FU,Cetuximab,Levofolinate,Oxaliplatin | Yes |
| CRC | Adenocarcinoma | 5-FU,Irinotecan,Levofolinate,Oxaliplatin | Yes |
| CRC | Adenocarcinoma | 5-FU,Irinotecan,Levofolinate,Panitumumab | Yes |
| CRC | Adenocarcinoma | 5-FU,Irinotecan,Levofolinate,Ramucirumab | Yes |
| CRC | Adenocarcinoma | Cisplatin,Etoposide | No |
| CRC | Adenocarcinoma | Cisplatin,Gemcitabine | No |
| CRC | Adenocarcinoma | Ipilimumab,Nivolumab | Yes |
| CRC | Adenocarcinoma | Irinotecan,S-1 | Yes |
| CRC | Adenocarcinoma | Levofolinate,UFT | Yes |
| CRC | Adenocarcinoma | Oxaliplatin,S-1 | Yes |
| CRC | Adenocarcinoma | S-1 | Yes |
| CUP | Adenocarcinoma | Carboplatin,Paclitaxel | Yes |
| CUP | Adenocarcinoma | S-1 | No |
| EC | Adenocarcinoma | 5-FU,Cisplatin | Yes |
| EC | Squamous Cell Carcinoma | 5-FU,Cisplatin | Yes |
| EC | Squamous Cell Carcinoma | 5-FU,Cisplatin,Docetaxel | Yes |
| EC | Squamous Cell Carcinoma | 5-FU,Oxaliplatin | Yes |
| EC | Squamous Cell Carcinoma | Nivolumab | Yes |
| EC | Squamous Cell Carcinoma | 5-FU,S-1 | No |
| EC | Squamous Cell Carcinoma | Carboplatin,Etoposide | No |
| GC | Adenocarcinoma | Oxaliplatin,S-1 | Yes |
| GC | Adenocarcinoma | 5-FU,Oxaliplatin | Yes |
| GC | Adenocarcinoma | Oxaliplatin,S-1,Trastuzumab | Yes |
| GC | Adenocarcinoma | Capecitabine,Oxaliplatin | Yes |
| GC | Adenocarcinoma | Capecitabine,Oxaliplatin,Trastuzumab | Yes |
| GC | Adenocarcinoma | Capecitabine,Cisplatin,Trastuzumab | Yes |
| GC | Adenocarcinoma | Cisplatin,S-1,Trastuzumab | Yes |
| GC | Adenocarcinoma | Docetaxel,S-1 | Yes |
| GC | Adenocarcinoma | Nab-Paclitaxel,Ramucirumab | Yes |
| GC | Adenocarcinoma | Cisplatin,S-1 | Yes |
| GC | Adenocarcinoma | Nivolumab | No |
| GC | Adenocarcinoma | S-1 | Yes |
| GC | Adenocarcinoma | 5-FU,Levofolinate,Oxaliplatin | Yes |
| GC | Adenocarcinoma | 5-FU,Levofolinate,Oxaliplatin,Trastuzumab | Yes |
| GC | Adenocarcinoma | Capecitabine,S-1 | No |
| GC | Adenocarcinoma | Cisplatin,Vaccine | Yes |
| GC | Adenocarcinoma | Oxaliplatin,S-1,Trastuzumab Deruxtecan | No |
| GC | Adenocarcinoma | Paclitaxel,Ramucirumab | Yes |
| GC | Undifferentiated Carcinoma | 5-FU,Oxaliplatin | Yes |
| GIST | GIST | Imatinib | Yes |
| HCC | Moderately Differentiated Hepatocellular Carcinoma | Lenvatinib | Yes |
| HCC | Moderately Differentiated Hepatocellular Carcinoma | Atezolizumab,Bevacizumab | Yes |
| HCC | Moderately Differentiated Hepatocellular Carcinoma | Sorafenib | Yes |
| HCC | Moderately Differentiated Hepatocellular Carcinoma | Cisplatin,Lenvatinib | No |
| HCC | Other | Atezolizumab,Bevacizumab | Yes |
| HCC | Poorly Differentiated Hepatocellular Carcinoma | Sorafenib | Yes |
| HCC | Poorly Differentiated Hepatocellular Carcinoma | Atezolizumab,Bevacizumab | Yes |
| HCC | Poorly Differentiated Hepatocellular Carcinoma | Lenvatinib | Yes |
| HCC | Undifferentiated Carcinoma | Lenvatinib | Yes |
| HCC | Well Differentiated Hepatocellular Carcinoma | Sorafenib | Yes |
| HCC | Well Differentiated Hepatocellular Carcinoma | Lenvatinib | Yes |
| HNC | Adenocarcinoma | 5-FU,Cisplatin,Pembrolizumab | Yes |
| HNC | Adenocarcinoma | Cisplatin,Docetaxel | Yes |
| HNC | Adenocarcinoma | Cisplatin,Doxorubicin | No |
| HNC | Adenocarcinoma | Docetaxel,Trastuzumab | Yes |
| HNC | Adenocarcinoma | Nivolumab | Yes |
| HNC | Adenocarcinoma | Pembrolizumab | Yes |
| HNC | Adenoid Cystic Carcinoma | Cisplatin,Docetaxel | Yes |
| HNC | Carcinoma Ex Pleomorphic Adenoma | Cisplatin,Docetaxel | Yes |
| HNC | Carcinoma Ex Pleomorphic Adenoma | Carboplatin,Paclitaxel | Yes |
| HNC | Mucoepidermoid Carcinoma | 5-FU,Cisplatin,Pembrolizumab | Yes |
| HNC | Mucoepidermoid Carcinoma | Carboplatin,Docetaxel | Yes |
| HNC | Salivary Duct Carcinoma | Cisplatin,Docetaxel | Yes |
| HNC | Salivary Duct Carcinoma | 5-FU,Carboplatin,Pembrolizumab | Yes |
| HNC | Salivary Duct Carcinoma | Carboplatin,Docetaxel | Yes |
| HNC | Squamous Cell Carcinoma | Pembrolizumab | Yes |
| HNC | Squamous Cell Carcinoma | Nivolumab | Yes |
| HNC | Squamous Cell Carcinoma | 5-FU,Carboplatin,Pembrolizumab | Yes |
| HNC | Squamous Cell Carcinoma | Carboplatin,Cetuximab,Paclitaxel | Yes |
| HNC | Squamous Cell Carcinoma | Cisplatin | Yes |
| HNC | Squamous Cell Carcinoma | Cetuximab,Paclitaxel | Yes |
| HNC | Squamous Cell Carcinoma | Cisplatin,Gemcitabine | Yes |
| HNC | Squamous Cell Carcinoma | 5-FU,Cetuximab,Cisplatin | Yes |
| HNC | Squamous Cell Carcinoma | 5-FU,Carboplatin,Cetuximab | Yes |
| HNC | Squamous Cell Carcinoma | 5-FU,Cisplatin | No |
| HNC | Squamous Cell Carcinoma | 5-FU,Pembrolizumab | No |
| HNC | Squamous Cell Carcinoma | Cetuximab Sarotalocan | Yes |
| HNC | Squamous Cell Carcinoma | Cisplatin,Docetaxel | No |
| HNC | Squamous Cell Carcinoma | Cisplatin,Pembrolizumab,S-1 | No |
| MCC | Merkel Cell Carcinoma | Avelumab | Yes |
| MEL | Acral Lentiginous Melanoma | Pembrolizumab | Yes |
| MEL | Acral Lentiginous Melanoma | Nivolumab | Yes |
| MEL | Acral Lentiginous Melanoma | Binimetinib,Encorafenib | Yes |
| MEL | Acral Lentiginous Melanoma | Ipilimumab,Nivolumab | Yes |
| MEL | Lentigo Maligna Melanoma | Ipilimumab,Nivolumab | Yes |
| MEL | Lentigo Maligna Melanoma | Pembrolizumab | Yes |
| MEL | Mucosal Melanoma | Ipilimumab,Nivolumab | Yes |
| MEL | Mucosal Melanoma | Nivolumab | Yes |
| MEL | Mucosal Melanoma | Pembrolizumab | Yes |
| MEL | Nodular Melanoma | Ipilimumab,Nivolumab | Yes |
| MEL | Nodular Melanoma | Nivolumab | Yes |
| MEL | Nodular Melanoma | Pembrolizumab | Yes |
| MEL | Nodular Melanoma | Dabrafenib,Trametinib | Yes |
| MEL | Other | Ipilimumab,Nivolumab | Yes |
| MEL | Superficial Spreading Melanoma | Dabrafenib,Trametinib | Yes |
| MEL | Superficial Spreading Melanoma | Ipilimumab,Nivolumab | Yes |
| MEL | Superficial Spreading Melanoma | Binimetinib,Encorafenib | Yes |
| MEL | Unclassified Cutaneous Melanoma | Dabrafenib,Trametinib | Yes |
| MEL | Unclassified Cutaneous Melanoma | Ipilimumab,Nivolumab | Yes |
| MEL | Unknown Primary Melanoma | Ipilimumab,Nivolumab | Yes |
| MEL | Unknown Primary Melanoma | Nivolumab | Yes |
| MEL | Unknown Primary Melanoma | Pembrolizumab | Yes |
| MEL | Uveal Melanoma | Ipilimumab,Nivolumab | Yes |
| MEL | Uveal Melanoma | Nivolumab,TACE | Yes |
| NEC | Mixed Adenoendocrine Carcinoma (MANEC) | Cisplatin,Irinotecan | Yes |
| NEC | Mixed Adenoendocrine Carcinoma (MANEC) | Cisplatin,Etoposide | Yes |
| NEC | Neuroendocrine Carcinoma: NEC (Large Cell or Small Cell Type) | Cisplatin,Etoposide | Yes |
| NEC | Neuroendocrine Carcinoma: NEC (Large Cell or Small Cell Type) | Cisplatin,Irinotecan | Yes |
| NEC | Neuroendocrine Carcinoma: NEC (Large Cell or Small Cell Type) | 5-FU,Streptozocin | Yes |
| NEC | Neuroendocrine Carcinoma: NEC (Large Cell or Small Cell Type) | Carboplatin,Etoposide | Yes |
| NEC | Neuroendocrine Carcinoma: NEC (Large Cell or Small Cell Type) | Everolimus | Yes |
| NEC | Other | Lanreotide | Yes |
| NET | Neuroendocrine Tumor: NET Grade 1 (NETG1) | Everolimus,Lanreotide | Yes |
| NET | Neuroendocrine Tumor: NET Grade 1 (NETG1) | Lanreotide | Yes |
| NET | Neuroendocrine Tumor: NET Grade 2 (NETG2) | Denosumab,Everolimus,Lanreotide | Yes |
| NET | Neuroendocrine Tumor: NET Grade 2 (NETG2) | Everolimus | Yes |
| NET | Neuroendocrine Tumor: NET Grade 2 (NETG2) | Everolimus,Lanreotide | Yes |
| NET | Neuroendocrine Tumor: NET Grade 2 (NETG2) | Lanreotide | Yes |
| NET | Neuroendocrine Tumor: NET Grade 2 (NETG2) | S-1,Streptozocin | Yes |
| NET | Neuroendocrine Tumor: NET Grade 2 (NETG2) | Sunitinib | Yes |
| NET | Well-differentiated PanNENs: PanNET G3 | 5-FU,Streptozocin | Yes |
| OV | Carcinosarcoma | Carboplatin,Paclitaxel | Yes |
| OV | Clear Cell Carcinoma | Bevacizumab,Carboplatin,Paclitaxel | Yes |
| OV | Clear Cell Carcinoma | Carboplatin,Paclitaxel | Yes |
| OV | Clear Cell Carcinoma | Doxorubicin | No |
| OV | Endometrioid Carcinoma | Carboplatin,Paclitaxel | Yes |
| OV | Endometrioid Carcinoma | Bevacizumab,Carboplatin,Paclitaxel | Yes |
| OV | Endometrioid Carcinoma | Carboplatin,Docetaxel | Yes |
| OV | High-Grade Serous Carcinoma | Carboplatin,Paclitaxel | Yes |
| OV | High-Grade Serous Carcinoma | Bevacizumab,Carboplatin,Paclitaxel | Yes |
| OV | High-Grade Serous Carcinoma | Carboplatin,Liposomal Doxorubicin | Yes |
| OV | High-Grade Serous Carcinoma | Bevacizumab,Carboplatin,Liposomal Doxorubicin,Paclitaxel | Yes |
| OV | High-Grade Serous Carcinoma | Carboplatin,Liposomal Doxorubicin,Olaparib | Yes |
| OV | Low-Grade Serous Carcinoma | Bevacizumab,Carboplatin,Paclitaxel | Yes |
| OV | Low-Grade Serous Carcinoma | Carboplatin,Paclitaxel | Yes |
| OV | Malignant | Carboplatin,Paclitaxel | Yes |
| OV | Mucinous Carcinoma | Bevacizumab,Carboplatin,Paclitaxel | Yes |
| OV | Mucinous Carcinoma | Carboplatin,Paclitaxel | Yes |
| OV | Seromucinous Carcinoma | Carboplatin,Paclitaxel | Yes |
| OV | Undifferentiated Carcinoma | Carboplatin,Paclitaxel | Yes |
| Others | Other | Carboplatin,Paclitaxel | Yes |
| Others | Other | Mitotane | Yes |
| PDAC | Acinar Cell Carcinoma | 5-FU,Irinotecan,Levofolinate,Oxaliplatin | Yes |
| PDAC | Acinar Cell Carcinoma | Gemcitabine,S-1 | Yes |
| PDAC | Adenocarcinoma | Gemcitabine,Nab-Paclitaxel | Yes |
| PDAC | Adenocarcinoma | 5-FU,Irinotecan,Levofolinate,Oxaliplatin | Yes |
| PDAC | Adenocarcinoma | 5-FU,Irinotecan,Oxaliplatin | Yes |
| PDAC | Adenocarcinoma | Irinotecan,Oxaliplatin,S-1 | No |
| PDAC | Adenocarcinoma | Gemcitabine | Yes |
| PDAC | Adenocarcinoma | S-1 | Yes |
| PDAC | Adenocarcinoma | Gemcitabine,Nivolumab,S-1 | No |
| PDAC | Adenocarcinoma | Gemcitabine,S-1 | Yes |
| PDAC | Adenocarcinoma | 5-FU,Docetaxel,Oxaliplatin | No |
| PDAC | Adenocarcinoma | 5-FU,Irinotecan | Yes |
| PDAC | Adenocarcinoma | 5-FU,Levofolinate,Oxaliplatin | Yes |
| PDAC | Adenocarcinoma | Gemcitabine,Irinotecan | No |
| PDAC | Adenocarcinoma | Gemcitabine,Irinotecan,Oxaliplatin,S-1 | No |
| PDAC | Adenocarcinoma | Irinotecan,Levofolinate,Nab-Paclitaxel,Oxaliplatin | No |
| PDAC | Adenocarcinoma | Irinotecan,Levofolinate,Oxaliplatin | Yes |
| PDAC | Adenocarcinoma | Irinotecan,Nab-Paclitaxel | No |
| PDAC | Adenocarcinoma | Irinotecan,S-1 | No |
| PDAC | Adenocarcinoma | Nab-Paclitaxel | No |
| PDAC | Adenosquamous Carcinoma | Gemcitabine,Nab-Paclitaxel | Yes |
| PDAC | Adenosquamous Carcinoma | 5-FU,Irinotecan,Levofolinate,Oxaliplatin | Yes |
| PDAC | Mucinous Cystadenocarcinoma, Noninvasive | Gemcitabine,Nab-Paclitaxel | Yes |
| PDAC | NoData | 5-FU,Irinotecan,Levofolinate,Oxaliplatin | Yes |
| PDAC | Other | Gemcitabine,Nab-Paclitaxel | Yes |
| PDAC | Other | 5-FU,Irinotecan,Levofolinate,Oxaliplatin | Yes |
| PDAC | Other | 5-FU,Irinotecan,Oxaliplatin | Yes |
| PRAD | Adenocarcinoma | Goserelin | Yes |
| PRAD | Adenocarcinoma | Abiraterone,Goserelin | Yes |
| PRAD | Adenocarcinoma | Leuprorelin | Yes |
| PRAD | Adenocarcinoma | Abiraterone | Yes |
| PRAD | Adenocarcinoma | Abiraterone,Leuprorelin | Yes |
| PRAD | Adenocarcinoma | Apalutamide,Degarelix | Yes |
| PRAD | Adenocarcinoma | Bicalutamide,Leuprorelin | Yes |
| PRAD | Adenocarcinoma | Goserelin,Leuprorelin | Yes |
| PRAD | Adenocarcinoma | Abiraterone,Degarelix | Yes |
| PRAD | Adenocarcinoma | Bicalutamide,Degarelix | Yes |
| PRAD | Adenocarcinoma | Enzalutamide | Yes |
| PRAD | Adenocarcinoma | Docetaxel | Yes |
| PRAD | Adenocarcinoma | Docetaxel,Goserelin | Yes |
| PRAD | Adenocarcinoma | Docetaxel,Leuprorelin | Yes |
| PRAD | Adenocarcinoma | Abiraterone,Goserelin,Leuprorelin | Yes |
| PRAD | Adenocarcinoma | Degarelix | Yes |
| PRAD | Adenocarcinoma | Enzalutamide,Leuprorelin | Yes |
| PRAD | Adenocarcinoma | Apalutamide,Goserelin | Yes |
| PRAD | Adenocarcinoma | Bicalutamide,Goserelin | Yes |
| PRAD | Adenocarcinoma | Abiraterone,Degarelix,Goserelin | Yes |
| PRAD | Adenocarcinoma | Apalutamide | Yes |
| PRAD | Adenocarcinoma | Bicalutamide | Yes |
| PRAD | Adenocarcinoma | Darolutamide,Leuprorelin | Yes |
| PRAD | Adenocarcinoma | Degarelix,Docetaxel | Yes |
| PRAD | Adenocarcinoma | Degarelix,Leuprorelin | Yes |
| PRAD | Adenocarcinoma | Abiraterone,Apalutamide,Leuprorelin | Yes |
| PRAD | Adenocarcinoma | Abiraterone,Degarelix,Leuprorelin | Yes |
| PRAD | Adenocarcinoma | Abiraterone,Docetaxel | Yes |
| PRAD | Adenocarcinoma | Bicalutamide,Goserelin,Leuprorelin | Yes |
| PRAD | Adenocarcinoma | Degarelix,Goserelin | Yes |
| PRAD | Adenocarcinoma | Enzalutamide,Goserelin | Yes |
| RCC | Clear Cell Renal Cell Carcinoma | Ipilimumab,Nivolumab | Yes |
| RCC | Clear Cell Renal Cell Carcinoma | Avelumab,Axitinib | Yes |
| RCC | Clear Cell Renal Cell Carcinoma | Axitinib,Pembrolizumab | Yes |
| RCC | Clear Cell Renal Cell Carcinoma | Sunitinib | Yes |
| RCC | Clear Cell Renal Cell Carcinoma | Pazopanib | Yes |
| RCC | Clear Cell Renal Cell Carcinoma | Atezolizumab | No |
| RCC | Clear Cell Renal Cell Carcinoma | Axitinib | Yes |
| RCC | Clear Cell Renal Cell Carcinoma | Nivolumab | No |
| RCC | Clear Cell Renal Cell Carcinoma | Sorafenib | No |
| RCC | NoData | Ipilimumab,Nivolumab | Yes |
| RCC | Other | Axitinib,Pembrolizumab | Yes |
| RCC | Other | Sorafenib | No |
| RCC | Papillary Renal Cell Carcinoma | Ipilimumab,Nivolumab | Yes |
| RCC | Papillary Renal Cell Carcinoma | Cabozantinib | Yes |
| RCC | Papillary Renal Cell Carcinoma | Avelumab,Axitinib | Yes |
| SBC | Adenocarcinoma | 5-FU,Oxaliplatin | Yes |
| SBC | Adenocarcinoma | 5-FU,Levofolinate,Oxaliplatin | Yes |
| SBC | Adenocarcinoma | Capecitabine,Oxaliplatin | Yes |
| SBC | Adenocarcinoma | Oxaliplatin,S-1 | Yes |
| THCA | Follicular Carcinoma | Lenvatinib | Yes |
| THCA | Medullary Carcinoma (C-Cell Carcinoma) | Lenvatinib | Yes |
| THCA | Papillary Carcinoma | Lenvatinib | Yes |
| THCA | Poorly Differentiated Carcinoma | Lenvatinib | Yes |
| THCA | Undifferentiated (Anaplastic) Carcinoma | Lenvatinib | Yes |
| THCA | Undifferentiated (Anaplastic) Carcinoma | Paclitaxel | Yes |
| UC | Invasive Urothelial Carcinoma | Cisplatin,Gemcitabine | Yes |
| UC | Invasive Urothelial Carcinoma | Carboplatin,Gemcitabine | Yes |
| UC | Invasive Urothelial Carcinoma | Pembrolizumab | Yes |
| UC | Invasive Urothelial Carcinoma | Cisplatin | No |
| UC | Invasive Urothelial Carcinoma | Gemcitabine,Nedaplatin | No |
| UC | Invasive Urothelial Carcinoma | Capecitabine,Oxaliplatin | No |
| UC | Invasive Urothelial Carcinoma | Carboplatin,Cisplatin,Gemcitabine | Yes |
| UCEC | Carcinosarcoma | Carboplatin,Paclitaxel | Yes |
| UCEC | Carcinosarcoma | Ifosfamide | Yes |
| UCEC | Clear Cell Carcinoma | Carboplatin,Paclitaxel | Yes |
| UCEC | Dedifferentiated Carcinoma | Carboplatin,Paclitaxel | Yes |
| UCEC | Endometrioid Carcinoma With Squamous Differentiation | Carboplatin,Paclitaxel | Yes |
| UCEC | Endometrioid Carcinomas | Carboplatin,Paclitaxel | Yes |
| UCEC | Endometrioid Carcinomas | Carboplatin,Docetaxel | Yes |
| UCEC | Endometrioid Carcinomas | Cisplatin,Doxorubicin | Yes |
| UCEC | Other | Carboplatin,Paclitaxel | Yes |
| UCEC | Serous Carcinoma | Carboplatin,Docetaxel | Yes |
| UCEC | Serous Carcinoma | Carboplatin,Paclitaxel | Yes |
| UCEC | Serous Carcinoma | Doxorubicin | Yes |
| UCEC | Undifferentiated Carcinoma | Carboplatin,Denosumab,Paclitaxel | Yes |
| UCEC | Undifferentiated Carcinoma | Carboplatin,Paclitaxel | Yes |

^a^ Cancer type abbreviations: AC, anal cancer; BC, breast cancer; BTC, biliary tract cancer; CESC, cervical cancer; CRC, colorectal cancer; CUP, cancer of unknown primary; EC, esophageal cancer; GC, gastric cancer; GIST, gastrointestinal stromal tumor; HCC, hepatocellular carcinoma; HNC, head and neck cancer; MCC, Merkel cell carcinoma; MEL, melanoma; NEC, neuroendocrine carcinoma; NET, neuroendocrine tumor; Others, other cancer types; OV, ovarian cancer; PDAC, pancreatic ductal adenocarcinoma; PRAD, prostate cancer; RCC, renal cell carcinoma; SBC, small bowel cancer; THCA, thyroid cancer; UC, urothelial carcinoma; UCEC, uterine corpus endometrial carcinoma.

^b^ Standard therapy was defined as a guideline-recommended first-line regimen based on clinical practice guidelines. “Yes” indicates the regimen matched the guideline-recommended first-line therapy; “No” indicates it did not; “Trial” indicates enrolment in a clinical trial (classified as guideline-concordant in all analyses).

**Online Resource 2. Cox proportional hazards regression for overall survival in metastatic colorectal cancer with molecular profile adjustment (Models 4a and 4b)**

| **Variable** | **HR^a^** | **95% CI^a^** | **p value** |
| --- | --- | --- | --- |
| **Model 4a^b^ (primary, n = 323; events = 202)** | | | |
| Specialist certification (Yes vs No [ref]) | 0.64 | 0.47–0.88 | 0.005** |
| Age (≥65 vs <65 [ref]) | 1.17 | 0.88–1.56 | 0.280 |
| Sex (Male vs Female [ref]) | 1.30 | 0.96–1.75 | 0.085 |
| Primary tumor side (Right vs Left [ref]) | 1.30 | 0.92–1.83 | 0.142 |
| Region (Kanto vs Other [ref]) | 1.09 | 0.80–1.49 | 0.584 |
| Trial participation (Trial vs Non-trial [ref]) | 1.75 | 0.70–4.39 | 0.231 |
| Molecular targeted therapy (Yes vs No [ref]) | 0.74 | 0.50–1.08 | 0.121 |
| No. metastatic organs: 2–3 (vs 1 [ref]) | 1.47 | 1.09–1.97 | 0.011* |
| No. metastatic organs: ≥4 (vs 1 [ref]) | 1.78 | 0.87–3.66 | 0.115 |
| RAS mutation (MT vs WT [ref]) | 1.53 | 1.12–2.11 | 0.008** |
| BRAF V600E (MT vs WT [ref]) | 2.12 | 1.38–3.27 | 0.001*** |
| **Model 4b^c^ (supplementary, n = 235; events = 148)** | | | |
| Specialist certification (Yes vs No [ref]) | 0.59 | 0.40–0.85 | 0.005** |
| Age (≥65 vs <65 [ref]) | 1.09 | 0.77–1.53 | 0.628 |
| Sex (Male vs Female [ref]) | 1.38 | 0.97–1.95 | 0.071 |
| Primary tumor side (Right vs Left [ref]) | 1.60 | 1.07–2.37 | 0.021* |
| Region (Kanto vs Other [ref]) | 1.06 | 0.73–1.54 | 0.762 |
| Trial participation (Trial vs Non-trial [ref]) | 1.52 | 0.54–4.28 | 0.426 |
| Molecular targeted therapy (Yes vs No [ref]) | 0.61 | 0.39–0.95 | 0.029* |
| No. metastatic organs: 2–3 (vs 1 [ref]) | 1.69 | 1.19–2.41 | 0.003** |
| No. metastatic organs: ≥4 (vs 1 [ref]) | 2.22 | 1.01–4.90 | 0.048* |
| RAS mutation (MT vs WT [ref]) | 1.50 | 1.04–2.16 | 0.031* |
| BRAF V600E (MT vs WT [ref]) | 1.94 | 1.18–3.20 | 0.009** |
| MSI-H (vs MSS [ref]) | 0.32 | 0.11–0.90 | 0.031* |

^a^ HR, hazard ratio; CI, confidence interval.

^b^ Model 4a (primary molecular-adjusted analysis): Model 3 additionally adjusted for RAS mutation and BRAF V600E status. Molecular profile data were obtained from routine clinical diagnostic test results performed at each participating institution and collected through the MONSTAR-SCREEN electronic case report form.

^c^ Model 4b (supplementary): Model 4a additionally adjusted for microsatellite instability (MSI) status. The reduced sample size reflects the proportion of patients with available MSI test results in the case report form.

* p < 0.05; ** p < 0.01; *** p < 0.001.

**Online Resource 3. E-value sensitivity analysis for unmeasured confounding**

| **Model** | **n** | **HR (95% CI)^a^** | **E-value^b^ (point estimate)** | **E-value^b^ (lower CI bound)** |
| --- | --- | --- | --- | --- |
| Model 1 (Basic + trial) | 358 | 0.72 (0.54–0.96) | 2.14 | 1.23 |
| Model 2 (+ Molecular targeted) | 358 | 0.74 (0.56–0.98) | 2.05 | 1.14 |
| Model 3 (+ Metastatic organs) | 354 | 0.74 (0.56–0.98) | 2.04 | 1.14 |
| Model 4a (+ RAS + BRAF V600E) | 323 | 0.64 (0.47–0.88) | 2.48 | 1.52 |
| Model 4b (+ MSI) | 235 | 0.59 (0.40–0.85) | 2.75 | 1.62 |

^a^ Hazard ratio (HR) and 95% confidence interval (CI) for specialist certification (Yes vs No [ref]) in each Cox proportional hazards model.

^b^ The E-value represents the minimum strength of association, on the hazard-ratio scale, that an unmeasured confounder would need to have with both the exposure (specialist certification) and the outcome (overall survival), conditional on the measured covariates, to fully explain the observed association. The E-value for the point estimate quantifies the minimum strength to explain the observed HR; the E-value for the lower CI bound quantifies the minimum strength to render the association no longer statistically significant. Calculated using the formula of VanderWeele and Ding (Ann Intern Med 2017;167:268–274).

**Supplementary Figure S1. Forest plot of hazard ratios for board certification and overall survival across all multivariable models in metastatic colorectal cancer.**

Forest plot of hazard ratios (HRs) and 95% confidence intervals for the association between JSMO board certification and overall survival in metastatic colorectal cancer across Models 1–4b. Models 1–3 are as described in Figure 4. Model 4a (n = 323) additionally adjusted for RAS and BRAF V600E mutation status; Model 4b (n = 235) additionally adjusted for microsatellite instability (MSI) status. Molecular profile data (RAS, BRAF V600E, and MSI) were obtained from routine clinical diagnostic test results performed at each participating institution and collected through the MONSTAR-SCREEN electronic case report form; sample size reductions in Models 4a and 4b reflect the proportion of patients with available test results for the respective markers.
